# Social Status and Emotional Competence in Bullying: A Longitudinal Study of the Transition From Kindergarten to Primary School

**DOI:** 10.3389/fpsyg.2022.817245

**Published:** 2022-04-29

**Authors:** Eleonora Farina, Carmen Belacchi

**Affiliations:** ^1^Department of Human Sciences for Education R. Massa, University of Milano-Bicocca, Milan, Italy; ^2^Department of Communication Sciences, Humanities and International Studies, University of Urbino Carlo Bo, Urbino, Italy

**Keywords:** bullying, emotional competence, social status, kindergarten, primary school, longitudinal approach

## Abstract

Moving on to a higher level of schooling represents a crucial developmental challenge for children: studies have shown that transitioning to a new school context can increase the perceived importance of peer acceptance, popularity, and adaptation to the new social environment. The aim of this study was to investigate simultaneously the influence of interpersonal variables (social status indices) and personal variables (empathy and understanding of emotions) on role-taking in bullying episodes (hostile, prosocial, victim, and outsider roles) from a longitudinal perspective. These variables were assessed on 41 children in their last year of kindergarten (t1) and in their 1st year of primary school (t2). The main longitudinal results showed that prosocial behaviors are more stable than hostile, victim, and outsider behaviors. Moreover, social preference—together with affective empathy—at t1 had a clear negative predictive effect on hostile roles at t2, while social preference had a positive effect on prosocial roles at t2. Social impact at t1 negatively predicted being a victim at t2. On the other hand, social preference at t2 was negatively predicted only by the victim role at t1. Social impact at t1 had a significant and negative effect on being victimized at t2 while was negatively predicted at t2 by the outsider at t1. Our study—even if exploratory—seems to highlight the existence of a specific, differentiate effect of two distinct social status indices on the participant role-taking in bullying episodes in the transitional period from kindergarten to primary school.

## Bullying in Pre-schoolers

Bullying is usually defined as a type of aggressive behavior that involves repeated physical and/or verbal attacks on a powerless individual (Salmivalli, 2010). Literature underlines the systemic nature of this phenomenon and the importance of the different figures who play a role in such situation. Participants to a bullying episode may react by adopting prosocial, hostile, or avoidant stances. The literature reports a number of attempts to model the full set of roles that may be played by bystanders (Salmivalli et al., 1996; Belacchi, 2008). Belacchi, following the seminal work of Salmivalli and colleagues (Salmivalli et al., 1996), assumed the existence of eight distinct roles in bullying episodes (Belacchi, 2008; Belacchi and Farina, 2010): *bully, victim, assistant* (provides direct assistance to the ringleader), *reinforcer* (indirectly supports the bully), *defender* (actively takes the part of the victim), *consoler* (comforts the victim), *mediator* (acts to reconcile bully and victim), and *outsider* (stands apart). These eight roles may be grouped into a four-factor structure: Prosocial macro-role (defender, consoler, and mediator), Hostile macro-role (bully, assistant, and reinforcer), Victim, and Outsider (Belacchi and Farina, 2010).

Over the past 30 years, an increasing volume of research has explored the features of the behaviors typical for each role and their individual and/or social correlates in school-age children, but only recently has the phenomenon been investigated in pre-schoolers (Monks et al., 2003, 2005; Belacchi and Farina, 2010, 2012; Vlachou et al., 2011, 2013; Farina and Belacchi, 2014). The tendency to assume certain roles in bullying episodes emerges at this early stage of development, but it tends to become stable only at later ages: for this reason, the study of young children’s roles in bullying is crucial. The few studies with pre-schoolers have identified psycho-social correlates like those reported for middle childhood and adolescence. For example, bullies are more likely to display insecure attachment and poor socio-emotional competence (Ortega Ruiz and Monks, 2005; Belacchi and Farina, 2012; Camodeca et al., 2015), poor inhibition skills (O’Toole et al., 2017), and are more prone to peer rejection (Wood et al., 2002), even if Perren and Alsaker (2006) found preschool bullies to be well embedded in their peer groups, with extensive friendship networks. Furthermore, preschool bullies tend to choose other aggressive peers as their friends (Dishion et al., 1994), which may—in turn—encourage aggressive behavior as a form of reciprocal adaptation. Studies focusing on victimized children describe them as physically weak, anxious, and sensitive, with poorer self-esteem and few friends (Olweus, 1993; Boulton and Smith, 1994; Perren and Alsaker, 2006). Camodeca et al. (2015) also found that victims displayed poor social competence but concluded that they did not suffer significant peer rejection. Finally, Belacchi and Farina (2012), using teacher reports, identified a negative correlation between being a victim and social desirability, which may be taken as indirect confirmation of victimized children’s poorer social adaptation skills.

Other studies that investigated the prosocial behavior of children in these situations (e.g., those who defend the victim or those who try to console him/her, Belacchi, 2008) have found that these children display more advanced socio-emotional skills. On the other hand, those who keep themselves out of situations have characteristics similar to those who enact hostile behaviors, showing poor social skills, and low levels of emotion comprehension and empathy (Monks et al., 2005; Belacchi and Farina, 2010, 2012; Camodeca et al., 2015).

A key to building up a full account of possible behaviors in bullying is investigating on their stability over time and their links with social status indices—besides with personal psychological dispositions—from early childhood. A recent cross-sectional study with Italian pre-schoolers highlighted significant associations among prosocial behavior, emotional competence, and social preference; on the other hand, hostile behaviors were directly linked with social impact and negatively with social preference; outsiders had low social impact among peers and poor emotional skills; and finally, children who experienced victimization more frequently were markedly the least preferred in the peer group (Farina and Belacchi, 2021).

## Bullying and Peer Social Status Over Time

The link between assumed roles in bullying episodes and peer social status goes through the investigation of roles’ stability over time. From studies on middle and late childhood, the role of victim is characterized by a less stable behavioral pattern than that of the bully (Kochenderfer-Ladd and Wardrop, 2001; Monks et al., 2003, 2021). Scholars have also suggested that the high number of young children who experience—but not stably—the role of victim may be more a function of social context than of individual characteristics: the low stability of dominance hierarchies in pre-schoolers’ social groups makes it easier for younger targets to avoid repeated aggression (Schäfer et al., 2005). In contrast, the general greater stability of the hostile behavior supports a view of it as mainly underpinned by personality and early socialization, in conjunction with social context. Regarding the stability of prosocial conducts in bullying episodes among pre-schoolers, Monks et al. (2003) found that the defending behavior was moderately stable.

Assuming that bullying is a group phenomenon, the maintenance of certain roles in such episodes is intertwined with relationships among peers in the same social context, like the classroom. The wide literature on peer social status offers different constructs to investigate on children’s relationships in the class. An important index refers to children *social preference* (calculated as the number of “likes” minus the number of “dislikes” assigned to a child by their peers; Coie et al., 1982), which can be seen as a sort of “positive popularity.” Anyway, the concept of popularity—including dimensions of power and prestige—is also close to individual visibility in the group (Cillessen and Marks, 2017), namely, children *social impact* (calculated as the sum of “likes” and “dislikes” received by one’s peers).

The literature suggests that, in late childhood, aggressive children are generally visible in the group, using this behavior to gain power, and therefore often perceived as popular, even if they are not liked (Cillessen and Mayeux, 2004; Cillessen and Rose, 2005). In contrast, socially preferred children often engage in prosocial interaction (Wentzel, 2003) during both childhood and adolescence (Hastings et al., 2007), while in the context of bullying episodes, they typically defend the victim (Monks et al., 2011).

Pellegrini and Long (2002) also underlined that the link between bullying and social relationships in school context can assume different characteristics in particular periods or events. The authors suggest that aggressive children make a higher use of bullying when they enter new social groups, for example, in the transition from primary to middle school, to achieve social dominance. This would lead to an establishment of a new hierarchy, followed by a decrease in bullying episodes and a stabilization of roles. Caravita and colleagues (Caravita et al., 2009) investigated on both individual characteristics—i.e., affective and cognitive empathy—and interpersonal variables—i.e., social preference and perceived popularity—influencing children’s bullying and defending behaviors. They collected data from primary and secondary school students, finding that bullying was negatively linked with social preference (calculated subtracting the like-least from the like-most nomination score) but, at the same time, positively associated with perceived popularity (score of nominations as popular) among boys and girls and in both age groups. Bullying was also negatively associated with affective empathy in the whole group and positively with cognitive empathy only among the older group. On the other hand, defending behavior resulted as positively associated with social preference but also with perceived popularity in the younger group. Defending was also positively associated with affective empathy, especially among children with high levels of social preference. The authors themselves admitted that the relationships between bullying/defending behavior and social status could be bi-directional rather than unidirectional and call for further investigations.

Studies at earlier ages are scarce, but some interesting findings can be helpful in clarifying certain patterns. To our knowledge, there is only one investigation by Lemerise et al. (1998) regarding peer social status in the transition from kindergarten to primary school: authors highlighted a general stability of acceptance and rejection in this period. Camodeca et al. (2015) identified a positive association between the defender role and general social competence, which in turn was both negatively associated with the other roles assessed (bully, supporter of the bully, outsider, and victim) and a negative predictor of social preference for preschool children who took the role of bully. This partially confirmed results from studies with older children, but important information on other positions in the peer group (e.g., visible—but not necessarily liked—children) is missing. To our knowledge, only a recent study explored the relationships of different roles in bullying with both social status and socio-emotional competence in kindergarteners (Farina and Belacchi, 2021): results highlighted a positive association of prosocial roles with social preference, emotion comprehension, and empathy; on the other hand, hostile behavior resulted negatively linked with social preference but positively with social impact; children who tend to avoid any involvement in a bullying episode showed low visibility (i.e., social impact) and emotional competences; and finally, frequently victimized children were the least preferred by peers. For these reasons, both social preference and social impact were included in the present study as interpersonal variables of interest.

Moving on to a higher level of schooling represents a crucial developmental challenge for children: studies have shown that transitioning to a new school context can increase the perceived importance of peer acceptance, popularity, and adaptation to the new social environment (Pellegrini, 2002; Juvonen and Ho, 2008). Longitudinal studies on the relationship between bullying roles and peer status have generally been conducted with older children, with a focus on the transition from primary to middle school or middle to high school. For example, Pouwels and colleagues studied how trajectory clusters of social status (social preference and perceived popularity) and behavior (direct aggression and prosocial behavior) from age 9 to age 14 predicted adolescents’ participant roles in bullying at age 16 and 17 (Pouwels et al., 2017). The main results highlighted that in adolescent bullies and followers, the “stable popular” trajectory cluster was the most present, whereas among the adolescent defenders the most important cluster was the “stable/average liked”; adolescent victims were mostly represented by the “unpopular/disliked” trajectory cluster, whereas no prevalent clusters were found for adolescent outsiders. These findings seem to confirm a predictive power of social status, in its dimension of popularity and preference, on the assumption of different roles in bullying episodes. To our knowledge, only a couple of studies have examined together bullying and social status during the transition from kindergarten to primary school. Ladd and Troop-Gordon (2003) found that aggression in last year of kindergarten predicted peer rejection in Grades 1 to 3, which in turn predicted loneliness and broader internalizing problems at age ten. Gooren et al. (2011), in a longitudinal study with a group of children in their last year of kindergarten and first year of primary school, found that peer rejection mediated the relationship between conduct issues and depressive symptoms. These links were similar for boys and girls and can be represented by a sort of “cascade effect” where conduct problems lead to poor peer experiences, which in their turn predict depression. These evidence highlight the possible bi-directional relationships between prosocial/hostile behaviors and peer social status: behaving aggressively make it less probable to be chosen by peers, which in its turn could reinforce an aggressive or withdrawal attitude. We therefore set out to assess the stability of bullying roles at an earlier passage, as children progress from kindergarten to primary school, as well as developments in the relationships between role-taking patterns, socio-emotional variables (i.e., emotion understanding and empathy), and social status. Given that the association between behavioral problems and peer rejection emerges at a very early age, we believe that it is crucial to study this links in greater depth and focus.

## Aims and Hypotheses

The aim of this study was to explore the influence of interpersonal variables (social status indices) and personal variables (empathy and emotion understanding) on children’s role-taking in bullying episodes, during the transition from kindergarten to primary school.

According to Salmivalli (2010), we could expect to find an increase in the frequency of hostile behaviors, as a means to acquire popularity in a new context. In addition, considering that social status influence looks wider and higher in hostile roles (Farina and Belacchi, 2021), we suppose these roles could be more sensitive to contextual conditions and—as a consequence—less stable in the transition to another school grade. Considering the scarcity of studies on possible social status changes during the transition between kindergarten and primary school, it is not possible to formulate precise hypotheses on social preference and social impact stability.

Regarding possible factors intervening on the stability of bullying roles over time, we expected that the tendency to assume certain roles in kindergarten would act as either risk or protective factors with respect to the tendency to behave aggressively at the later time. Following Schäfer et al. (2005), we hypothesized that children’s hostile and prosocial behaviors at t1 (kindergarten) would predict the frequency they enact the same behaviors at t2 (primary school). In contrast, considering the ambiguous features and low stability of victim and outsider role-taking in pre-schoolers (Belacchi and Farina, 2010, 2012), we did not expect to find any predictive power of these roles. According to literature (Camodeca et al., 2015; Farina and Belacchi, 2021), we hypothesized that social preference and social impact at t1 would predict hostile role-taking at t2, in negative and positive directions, respectively. Furthermore, based on the assumption that emotional competence fosters the development of positive relationships and prosocial behavior, we expected that personal factors (i.e., empathy) at t1 would predict a tendency to assume prosocial roles at t2. Following the hypothesized bi-directionality of such relationships (Caravita et al., 2009), the longitudinal design of the present study allows us to test the possibility that peer social status could be predicted by children roles in bullying together with their personal socio-emotional competences: even if in literature there are some valid results examining an association among these variables, the direction of such relationships is still unclear (Camodeca et al., 2015; Farina and Belacchi, 2021). Starting from these studies, we hypothesize that social impact could be predicted by hostile roles at t1, whereas personal socio-emotional competence would positively predict social preference at t2.

## Materials and Methods

### Participants

A sample of 41 children was tested at the end of their last year in kindergarten and then re-tested one year later, after entering primary school. An *a priori* power analysis has been used to estimate the sample size (using GPower 3.1; Faul et al., 2007). With an alpha = 0.05 and power = 0.85, the projected sample size needed to detect a medium effect size (*f* = 0.50) is approximately of at least *N* = 38 for each group (differences between two dependent means. Matched pairs); therefore, the study meets these power requirements. This group was composed of 17 boys and 24 girls (Mean age at t1 = 64.73 months, *SD* = 3.17; Mean age at t2 = 80.56 months, *SD* = 3.18) from seven kindergarten classes (along with their kindergarten class teachers) at t1. These children at t2 were distributed among four different first grade classes. Both in kindergarten and in primary school, one teacher per class completed the teacher-report instruments. Therefore, different informants were included in the design, in order to study the main variables. Written informed parental consent, as well as oral informed child consent, was obtained for all children. The study was conducted in accordance with the ethical standards laid down in the 1964 Declaration of Helsinki, and fulfilled the ethical standard procedures recommended by the Italian Association of Psychology.

### Instruments and Scoring

*Participant role questionnaire—PRQ (teacher version)* was used to measure the four macro-roles in bullying episodes (Belacchi and Farina, 2010). It is composed of 24 items and requires the teachers to indicate how frequently they observed specific prosocial and hostile behaviors in their children, using a 5-point Likert scale (never =1; always = 5). The validation of PRQ (Belacchi, 2008; Belacchi and Farina, 2010) highlighted that the eight roles are articulated in four macro-roles: hostile (bully, assistant, and reinforcer), prosocial (defender, consoler, and mediator), plus victim, and outsider. Children received an average score for each of these macro-roles. Preliminary reliability analyses were conducted for the items measuring: prosocial roles (*N* = 9), hostile roles (*N* = 9), victim, and outsider (*N* = 3 each). Cronbach’s α values were highly satisfactory for the prosocial roles (*α* = 0.88), hostile roles (*α* = 0.91), satisfactory for victim (*α* = 0.73), and almost satisfactory for outsider (*α* = 0.52). This last result is in line with previous findings (Belacchi and Farina, 2010).

*Empathic Responsiveness Scale—ERS* is a modified and abridged version of the Interpersonal Reactivity Index (Davis, 1980; Belacchi and Farina, 2012) and was used to assess cognitive and affective empathy. It comprised eight items, four per 2 sub-scales: perspective taking and empathic concern. Teachers were asked to rate the frequency with which they had observed empathic behavior in each of their students on a 5-point Likert scale (never =1 and always = 5). For each child empathic concern, perspective taking and global empathy scores were calculated. Reliability analyses were carried out for the global empathy scale (*N* = 8) and each of the two sub-scales: empathic concern (*N* = 4) and perspective taking (*N* = 4). The Cronbach’s *α* values obtained confirmed excellent reliability for the global scale (*α* = 0.82) and satisfactory reliability for both empathic concern (*α* = 0.75) and perspective taking (*α* = 0.77).

*Test of Emotion Comprehension—TEC* (Pons and Harris, 2000), in its Italian standardized version (Albanese and Molina, 2008), was used to evaluate emotion comprehension. This instrument consists of a set of 23 cartoon scenarios, testing nine different aspects of emotion understanding. For each scenario, the child is asked to attribute an emotion to the main character by pointing to one of the four depicted options. 1 point was given to each correct answer and 0 for each wrong answer, yielding an overall Emotion Understanding score (range 0–9) and three partial scores (0–3), for external, mental, and reflective sub-dimensions.

*Peer nominations* were used to assess social status, in its dimensions of social preference and social impact. Following McCandless and Marshall’s (1957) method of assessing social status among pre-schoolers, children were shown pictures of their schoolmates and asked to select three children they liked to play with and three children they did not like to play with. For each child, the frequencies of “likes” and “dislikes” received were calculated separately (min 0; max = *n*-1, where *n* is the number of children for each class). We followed the scoring procedure recommended by Coie et al. (1982): each participant’s (class-standardized) “liked” (zL) and “disliked” (zD) values were summed (zL + zD) to obtain a social impact index, and the difference between them calculated (zL – zD) to obtain a social preference index. Social impact and social preference were again class-standardized.

### Procedure and Statistical Analyses

Children were individually administered the TEC and social status interview in a quiet room in their school. Each session lasted a maximum of 30 min. The teachers were given two weeks to complete and return the PRQ and ERS for each of their children, after they had received detailed instructions and any necessary clarification from the researcher. Notably, they were all briefed on the conventional definition of bullying among peers (Genta et al., 1996). *T*-test, correlational, and hierarchical regression analyses (stepwise method) were conducted in order to explore the longitudinal relationships among the variables. The regressions were conducted testing two possible directions of predictability: the first with bullying roles and the second with social status indices as dependent variables.

## Results

### Correlations Among Variables at t1 and t2

We preliminarily performed correlational analyses among all variables at t1 and t2. The descriptive correlational pattern showed in Table 1 guided our following inferential analyses, mainly focused on longitudinal relationships between personal and interpersonal variables and role-taking.

**Table 1:** Correlational pattern among variables at t1 and t2 (Pearson’s *r*).

	Tec external t2	Tec mental t2	Tec reflective t2	Empathic concern t2	Perspective taking t2	Prosocial t2	Hostile t2	Victim t2	Outsider t2	Social preference t2	Social impact t2
Tec external t1	0.328*	0.024	0.034	0.248	0.235	0.240	−0.003	−0.047	−0.113	0.192	−0.050
Tec mental t1	0.055	−0.107	0.247	0.296	0.118	0.205	0.011	0.011	−0.139	−0.114	0.060
Tec reflective t1	0.128	−0.039	0.364*	−0.052	0.102	0.031	0.016	−0.069	0.055	−0.011	−0.093
Empatic concern t1	0.139	0.106	−0.194	−0.006	0.215	−0.130	−0.470**	−0.345*	0.173	0.157	−0.366*
Perspective taking t1	0.156	0.220	−0.195	−0.044	0.232	−0.055	−0.331*	−0.236	−0.279	0.164	−0.343*
Prosocial t1	0.098	0.229	−0.297	0.045	0.054	0.084	−0.273	−0.256	0.182	0.133	−0.027
Hostile t1	−0.053	−0.237	0.056	−0.060	−0.105	−0.215	0.268	0.279	−0.216	−0.217	0.247
Victim t1	−0.324*	−0.279	−0.174	−0.317*	−0.165	−0.406**	0.128	0.225	0.101	−0.494***	−0.155
Outsider t1	−0.050	−0.146	−0.073	0.155	0.008	0.003	0.141	0.284	−0.252	−0.185	−0.071
Social Preference t1	0.269	0.118	0.159	0.476**	0.376*	0.358*	−0.389*	−0.305	−0.097	0.461**	0.049
Social Impact t1	0.118	0.119	0.108	−0.026	0.002	0.074	0.048	−0.213	−0.099	0.018	0.265

* *p < 0.05*;

** *p < 0.01*;

*** *p < 0.001*.

### Stability of Participant Roles and Social Status

A paired *t*-test analysis was conducted to evaluate change or stability of roles over time. T-indices were significant for hostile role, victim, and outsider. Observation of the mean scores suggests that there was a significant decrease in adoption of all the abovementioned roles. Nevertheless, non-significant paired sample correlations indicated a different subject ranking from t1 to t2. The *T*-test was not significant for the prosocial role. Regarding social status, we can observe a general increase of both indices, but the *T*-test did not indicate a statistical significance. Paired sample correlation highlighted a similar subject ranking for social preference from t1 to t2 (see Table 2).

**Table 2:** Mean scores (and standard deviations) on participant roles and social status measures at t1 and t2, *T*-test, and paired correlations.

		T1	T2	T	*p Value*	Cohen’s *d*	Pearson’s *r*
Participant roles	*Prosocial*	2.25 (0.50)	2.11 (0.85)	0.953	0.346	0.40	0.084
	*Hostile*	2.09 (0.93)	1.35 (0.48)	5.091	0.001	1.13	0.268
	*Victim*	2.15 (0.84)	1.13 (0.32)	7.841	0.001	1.71	0.225
	*Outsider*	2.79 (0.57)	1.71 (0.82)	6.195	0.001	2.68	-0.252
Social status	*Social Preference*	0.01 (1.01)	0.12 (1.13)	-0.700	0.488	1.12	0.461***
	*Social Impact*	-0.01 (0.99)	0.20 (0.94)	-1.116	0.271	1.18	0.265

*** *p < 0.001*.

### Predictors of Participant Roles at t2

Hierarchical regressions were conducted to test the predictive power of both individual and interpersonal variables in the last year of kindergarten on the tendency to assume different roles at the beginning of primary school. Specifically, regression on roles at t2 tested the predictive power of the roles themselves at t1 (first step) and social preference, social impact, empathic concern, perspective taking, external, mental, and reflective dimensions of emotion understanding at t1 (second step). The prosocial role was positively predicted by social preference only (R^2^ = 0.128; *β* = 0.358; t = 2.397; *p* < 0.05), whereas the hostile role was negatively predicted by both empathic concern (R^2^ = 0.221; *β* = −0.403; *t* = − 2.903; *p* < 0.01) and social preference (R^2^ = 0.305; ΔR^2^ = 0.098; *β* = −0.298; t = −2.147; *p* < 0.05). The victim too was negatively predicted by empathic concern (R^2^ = 0.119; *β* = −0.460; *t* = −3.074; *p* = 0.004) and social impact (R^2^ = 0.236; ΔR^2^ = 0.117; *β* = −0.360; *t* = −2.405; *p* < 0.05). No significant predictors were found for the outsider.

### Predictors of Social Preference and Social Impact at t2

In order to explore both personal and interpersonal variables during the last year of kindergarten as possible predictors of social status indices at the beginning of primary school, we conducted hierarchical multiple regressions on social preference and social impact at t2, with social preference and social impact at t1 (first step), participant roles, emotion understanding, empathic concern, and perspective taking at t1 (second step), as independent variables. Social preference at t2 was predicted by social preference at t1 (R^2^ = 0.224; *β* = 0.473; *t* = 3.353; *p* = 0.002), but this relationship lost its significance after the role-taking patterns were entered at the second step, with variance now only negatively predicted by the role of victim at t1 (R^2^ = 0.302; *β* = −0.341; *t* = −2.070; *p* < 0.05). The negative predictors of social impact at t2 were empathic concern (R^2^ = 0.120; *β* = −0.511; *t* = −3.098; p = 0.004) and outsider role (R^2^ = 0.208; ΔR^2^ = 0.088; *β* = −0.339; *t* = −2.055; *p* < 0.05).

**Figure 1:**
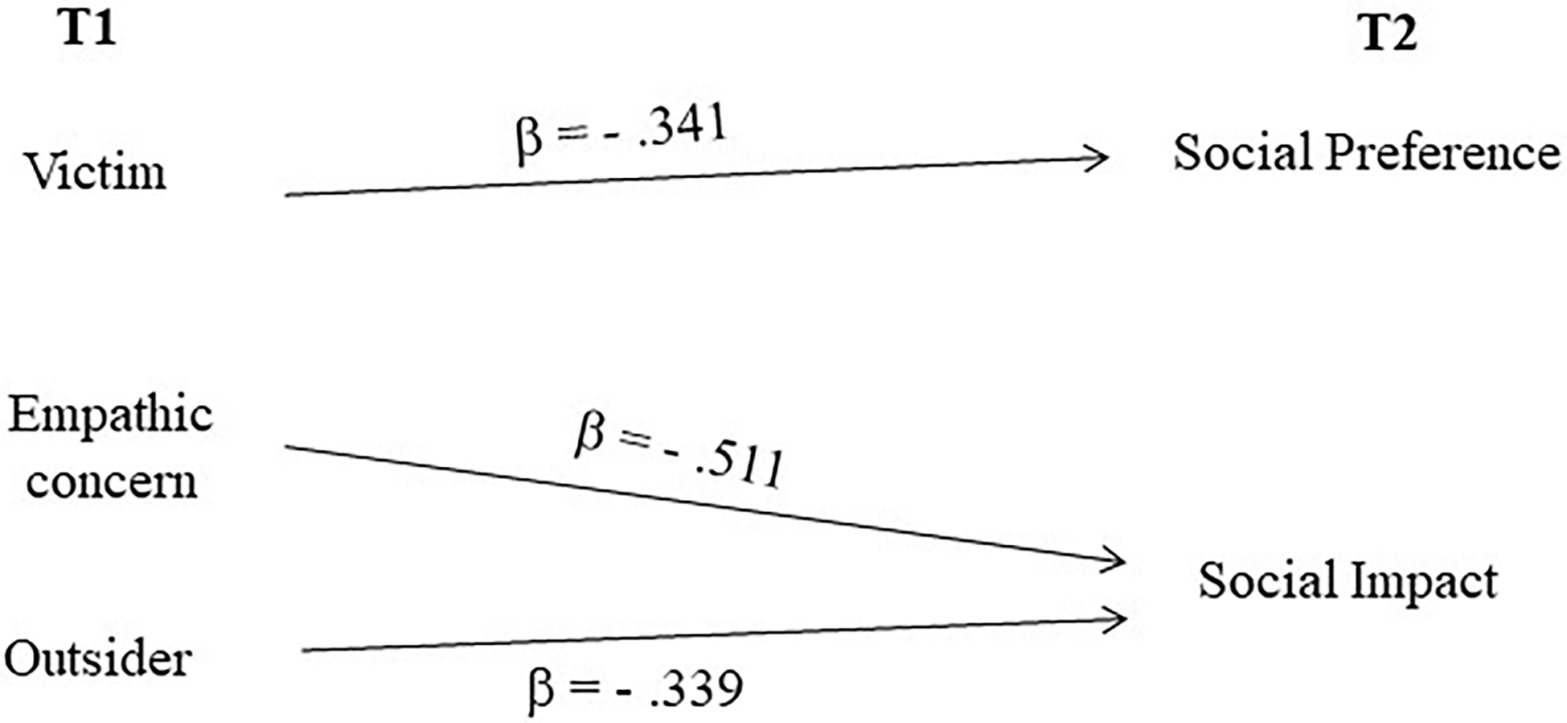
Significant predictors at t1 of participant roles at t2 (regression analyses).

**Figure 2:**
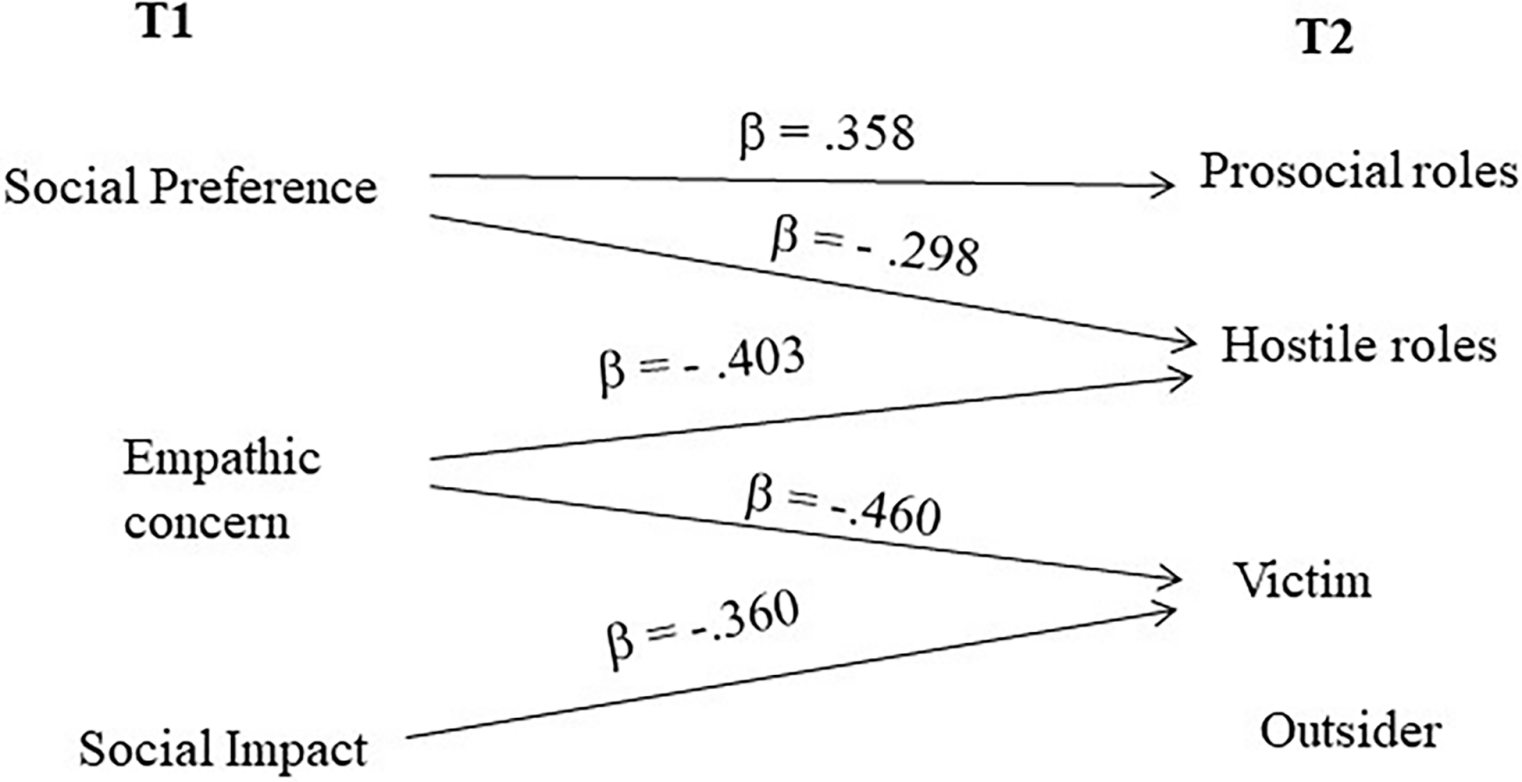
Significant predictors at t1 of social status indices at t2 (regression analyses).

A graphical summary of the regression analyses is shown in Figures 1, 2, in which the significant predictors (variables measured at t1) of roles (Figure 1) and social status at t2 (Figure 2) are shown.

## Discussion

The aim of this study was to investigate simultaneously the influence of interpersonal variables (social status indices) and personal variables (empathy and understanding of emotions) on role-taking in bullying episodes, from a longitudinal perspective. Such research design aimed also at clarifying and corroborating some recent cross-sectional findings highlighting a pattern of inter-relationships among such variables. In particular, literature showed an association between being liked by peers, behaving prosocially, and showing good emotional competence on one hand, and being visible, behaving aggressively, and being less emotionally competent, on the other hand. Furthermore, poor social preference was associated with higher victimization and poor social impact along with poor emotional skills with the tendency to be detached from the bullying situation (Farina and Belacchi, 2021). The novel contribution of the present work is its focus on preschool children, studying longitudinally the abovementioned associations during the crucial transition from kindergarten to primary school. Differently from the results of Salmivalli et al. (1998) who found a substantial stability in all roles in a longitudinal study with preadolescents, our results showed instability over time, above all for hostile roles, but also—and with a larger effect size (see Cohen’s *d* in Table 2)—for victim and outsider. Scores for all these three roles changed significantly from t1 to t2, registering a decrease and revealing a possible deep influence by a changed context. Furthermore, during this particular transition, children may perceive complying with the rules as the best way to earn acceptance: they may not feel the need to rebel against the social norms, represented by the adult, as a means to enhance their power in the peer group (Schäfer et al., 2005). Thus, it is possible that in the new context of primary school, children tend to inhibit hostile behavior in order to be accepted. Prosocial behavior maintains a certain stability, even if the non-significant paired correlations revealed a different subject ranking over time. This evidence suggests the opportunity to study specific profiles among prosocial roles (this could be possible using the PRQ, which distinguishes between defending, consoling, and mediating behaviors, but in this study, the small number of participants did not support analyses at this level of detail). Furthermore, such correlational indices—non-significant for the other roles, too—could be also interpreted as a confirmation of general low stability of participants’ roles at a very young age (Monks et al., 2003): an important and favorable characteristic for preventive interventions.

Observing the stability of peer social status, our results confirmed a general tendency to maintain levels of social acceptance and rejection during this transition, but paired correlations highlighted an individual preservation of social preference levels: children with high social preference at kindergarten seemed to be highly preferred at primary school, too.

Regarding the aim of clarifying the predictive role of interpersonal and personal variables in prosocial and hostile role-taking, our results overall confirm our hypotheses, supporting the abovementioned evidence of a cross-sectional study on children of the same ages (Farina and Belacchi, 2021). We found that earlier social preference positively predicted the adoption of prosocial roles in the new social context, confirming that, in our sample, being previously liked acted as a protective factor against engaging in aggressive behaviors, as reported for older children and adolescents (Caravita et al., 2009; Pouwels et al., 2017). On the other hand, the negative predictive power of social preference on hostile roles is in keeping with the findings of Caravita et al. (2009): perceived low social preference could therefore be a threat to personal social status, leading children to enact aggressive behaviors as a form of self-defense. Furthermore, hostile roles at t2 were predicted by low levels of empathic concern, showing the interdependence between internal and contextual dimensions in hostile role assumption. Interestingly, adopting the role of victim is most strongly predicted by low visibility among peers and poor empathic skills: this is in line with studies describing victimized children as poor in socio-emotional competence and friends (Vlachou et al., 2011; Belacchi and Farina, 2012; Farina and Belacchi, 2021). Such a framework is also compatible with the results of our regressions on social status indices: being victimized may represent a longitudinal risk factor for not being chosen by peers (social preference at t2). On the other hand, those who have a history of staying away from conflict (outsider role) or of displaying low levels of empathic concern appear to go unnoticed by their peers (low social impact). As argued above, in the primary school context, the adult’s authority still holds considerable sway and children may not need to reinforce their hostile or aggressive behaviors to gain popularity; therefore, the decrease in hostile role-taking may be to some degree a function of the new context (Schäfer et al., 2005). The fact that social preference at t1 predicted positively prosocial roles and negatively hostile ones at t2 confirms high social preference as a protective factor with respect to hostile role-taking and a stimulus to behave prosocially. Furthermore, considering the stability of social preference detected in this study, it is possible to suppose a sort of virtuous circle supported by the intertwining relationships between being liked and being prosocial. In any case, although some studies reported in the literature suggest that, up to adolescence, being popular (in terms of visibility), and being chosen often coincides (see Pouwels et al., 2017), the present research shows that in early childhood, these two variables are clearly distinct and wield different influences on role-taking during bullying.

Taken together, the results seem to suggest that prosocial role-taking stabilizes earlier than hostile role-taking. This may be due to the fact that personal positive dispositions are more strongly involved in prosocial behaviors than are social status indices (Farina and Belacchi, 2021). In turn, our longitudinal results showed that probably, already at an early age, a virtuous circle is established between prosocial behavior and preference by peers. Conversely, the relationships among hostile roles and social status variables could create—in perspective—a vicious circle. The establishment and evolution over time of these possible circular relationships should be investigated in greater depth.

In conclusion, we underline that the current results are still exploratory and call for further confirmation, for a number of reasons. Firstly, participants’ cognitive and verbal abilities were not assessed, although at this age such factors can be implicated in children’s social behavior. Neither did we control for other variables, such as family socio-economic status, which is often included in the design of studies in this area. Again, this variable should be considered in future research. Furthermore, a longitudinal survey should ideally be conducted with a higher number of participants and over a longer time. In this study, the subjects were re-tested after only one year, albeit during a very important step of their socio-relational development. Finally, it must be taken into account that the detected change in roles and social status may partly depend on the fact that the informants (teachers and peers) changed from t1 to t2.

Despite these limitations, we think it is clear that early identification of the personal and interpersonal variables associated with bullying dynamics can help prevent them from worsening and from negatively impacting on children’s development (e.g., Vlachou et al., 2011). Furthermore, the differential contributions of personal and interpersonal factors to prosocial versus hostile role-taking can inform intervention programs designed to address and promote both personal attitudes and group dynamics (Caravita et al., 2009). Such programs should focus, on the one hand, on the development of personal socio-emotional competences as protective factors, and on the other, on the observation and modulation of interpersonal relationships in the classroom.

## Data Availability Statement

The raw data supporting the conclusions of this article will be made available by the authors, without undue reservation.

## Ethics Statement

Ethical review and approval was not required for the study on human participants in accordance with the local legislation and institutional requirements. Written informed consent to participate in this study was provided by the participants’ legal guardian/next of kin.

## Author Contributions

EF contributed to design the study, collected, interpreted, discussed the data, and wrote and revised the manuscript. CB contributed to design the study, interpreted and discussed the data, and wrote and revised the manuscript. All authors contributed to the article and approved the submitted version.

## Funding

This work was supported by a grant from the University of Milano-Bicocca assigned to EF for the year 2020.

## Conflict of Interest

The authors declare that the research was conducted in the absence of any personal, professional, or financial relationships that could be construed as a potential conflict of interest.

## Publisher’s Note

All claims expressed in this article are solely those of the authors and do not necessarily represent those of their affiliated organizations, or those of the publisher, the editors and the reviewers. Any product that may be evaluated in this article, or claim that may be made by its manufacturer, is not guaranteed or endorsed by the publisher.
